# Climatic niche shift predicts thermal trait response in one but not both introductions of the Puerto Rican lizard *Anolis cristatellus* to Miami, Florida, USA

**DOI:** 10.1002/ece3.263

**Published:** 2012-07

**Authors:** Jason J Kolbe, Paul S VanMiddlesworth, Neil Losin, Nathan Dappen, Jonathan B Losos

**Affiliations:** 1 Museum of Comparative Zoology and Department of Organismic and Evolutionary Biology, Harvard University26 Oxford St., Cambridge, Massachusetts 02138; 2 Department of Ecology and Evolutionary Biology, University of California Los Angeles621 Charles E. Young Dr. South, Los Angeles, California 90095; 3 Department of Biology, University of Miami1301 Memorial Dr., Coral Gables, Florida 33124

**Keywords:** Acclimation, critical thermal minimum, low-temperature tolerance, phenotypic plasticity, species distribution modeling

## Abstract

Global change is predicted to alter environmental conditions for populations in numerous ways; for example, invasive species often experience substantial shifts in climatic conditions during introduction from their native to non-native ranges. Whether these shifts elicit a phenotypic response, and how adaptation and phenotypic plasticity contribute to phenotypic change, are key issues for understanding biological invasions and how populations may respond to local climate change. We combined modeling, field data, and a laboratory experiment to test for changing thermal tolerances during the introduction of the tropical lizard *Anolis cristatellus* from Puerto Rico to Miami, Florida. Species distribution models and bioclimatic data analyses showed lower minimum temperatures, and greater seasonal and annual variation in temperature for Miami compared to Puerto Rico. Two separate introductions of *A. cristatellus* occurred in Miami about 12 km apart, one in South Miami and the other on Key Biscayne, an offshore island. As predicted from the shift in the thermal climate and the thermal tolerances of other *Anolis* species in Miami, laboratory acclimation and field acclimatization showed that the introduced South Miami population of *A. cristatellus* has diverged from its native-range source population by acquiring low-temperature acclimation ability. By contrast, the introduced Key Biscayne population showed little change compared to its source. Our analyses predicted an adaptive response for introduced populations, but our comparisons to native-range sources provided evidence for thermal plasticity in one introduced population but not the other. The rapid acquisition of thermal plasticity by *A. cristatellus* in South Miami may be advantageous for its long-term persistence there and expansion of its non-native range. Our results also suggest that the common assumption of no trait variation when modeling non-native species distributions is invalid.

## Introduction

Understanding how populations respond to global environmental change is one of the most important and daunting challenges facing applied biologists (Sakai et al. 2001; Parmesan 2006). Species invasions offer unprecedented opportunities for understanding the ecological and evolutionary responses of populations to rapidly changing environments. Invaders inevitably face novel conditions during their establishment and spread, such as the addition or loss of interacting species (e.g., Strauss et al. 2006) or altered climatic conditions (e.g., Broennimann et al. 2007). Thus, a key issue in invasion biology is whether invaders show a phenotypic response to this environmental change, and if so, whether this is accomplished by adaptation, phenotypic plasticity, or a combination of both (Lee 2002; Richards et al. 2006; Ghalambor et al. 2007). Determining whether the phenotypic changes experienced by invading populations contribute to invasion success is fundamental to understanding current biological invasions and predicting future ones (Whitney and Gabler 2008).

Phenotypic change in natural populations can be rapid, particularly in cases of human disturbance (Stockwell et al. 2003; Hairston et al. 2005; Hendry et al. 2008). In most instances, however, the relative contribution of adaptation and phenotypic plasticity to the observed phenotypic change is not known (Ghalambor et al. 2007; Hendry et al. 2008). Studies that evaluate these mechanisms by combining observations of phenotypic change with common garden studies, controlled breeding designs, or plasticity experiments are rare, and often do not make a clear hypothesis of an adaptive relationship (e.g., a trait-environment correlation) or quantify environmental change. Despite a lack of understanding of the causal mechanisms underlying rapid phenotypic change in most systems, species invasions provide some examples of both adaptive and plastic phenotypic responses to changing environmental conditions during invasion (e.g., Huey et al. 2000; Lee 2002; Kolbe et al. 2010). Invasive species make excellent models for studying rapid phenotypic change because we often know or can reconstruct the times, locations, and sources of introductions (Kolbe et al. 2004). Such knowledge allows us to quantify environmental shifts and the native-range source population provides the baseline for detecting phenotypic changes.

Species distribution modeling (SDM, or ecological/environmental niche modeling) has recently found wide application in invasion biology for predicting non-native geographic ranges, particularly for risk assessment (e.g., Peterson 2003; Thuiller et al. 2005; Elith et al. 2010). While niche modeling is a potentially powerful tool for predicting non-native ranges, there are a number of problems that may reduce the accuracy of such predictions, including extrapolation to novel environmental space, lack of niche conservatism (i.e., genetic and phenotypic change in the fundamental niche), and nonequilibrium conditions due to ongoing spread (Kearney 2006; Jeschke and Strayer 2008; Elith et al. 2010). Making accurate predictions is certainly an important goal; however, a related use of SDM in invasion biology is detecting shifts in the climatic conditions occupied by a species from its native to non-native ranges (e.g., Broennimann et al. 2007; Mandle et al. 2010). In this context, extrapolation to novel environments and ongoing range expansion may be indicators of shifting environmental conditions during invasion, which could drive adaptation or plastic responses. Thus, SDM can be a tool for predicting phenotypic change by quantifying shifts in climatic conditions from the native to non-native range, exposing subtle differences among non-native populations, and isolating variables that reflect exposure to novel environmental conditions. Only a few studies have combined SDM and measures of phenotypic variation (e.g., Terblanche et al. 2006; Wright et al. 2006; Kolbe et al. 2010), and to our knowledge no study has used SDM to detect a climatic niche shift during invasions in order to predict phenotypic responses to the novel environment, and then to test those predictions with field and experimental data on phenotypic variation.

**Figure 1:**
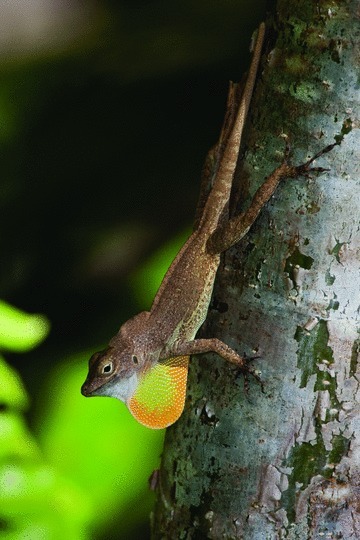
A male *Anolis cristatellus* from Miami with its dewlap extended.

We used the introduction of the tropical lizard *Anolis cristatellus* (Fig. 1) from its native range of Puerto Rico to Miami, Florida, USA to test for a thermal niche shift and divergence in thermal tolerance between ranges. An advantage of this study system is its well-characterized introduction history, including independent introductions to at least two locations in South Florida. *Anolis cristatellus* has been introduced to Key Biscayne, where it was first detected in 1975 (Schwartz and Thomas 1975; Bartlett and Bartlett 1999) and South Miami, where it was first detected in 1976 (Wilson and Porras 1983; Bartlett and Bartlett 1999). Phylogeographic analysis of mtDNA haplotypic variation sampled from the introduced and native ranges revealed two geographically and genetically distinct native-range source populations (Kolbe et al. 2007). The Key Biscayne population originated in the San Juan area, whereas the South Miami population is derived from the Agua Claras/Ceiba area of northeast Puerto Rico. Neither population has spread outside of the Miami metropolitan area over the past 35 years. The Key Biscayne population is separated from the mainland population by a bridge to Virginia Key and then a causeway to the mainland, and these two initial sites of introduction are ∼12 km apart across Biscayne Bay.

The thermal biology of lizards in the genus *Anolis* (or anoles) has been well studied (reviewed in Losos 2009). Tropical lizards and other ectotherms are predicted to lack temperature acclimation ability (Janzen 1967) and they should not tolerate temperatures as low as those tolerated by their temperate counterparts (Tsuji 1988; Rogowitz 1996; Ghalambor et al. 2006; Huey et al. 2009). For example, previous studies of the native anole species in the southeastern United States, *A. carolinensis,* suggest that it can acclimate to low temperatures (Kour and Hutchinson 1970; Wilson and Echternacht 1987). By contrast, evidence for similar plasticity in tropical *Anolis* species is lacking, and native-range *A. cristatellus* had reduced short-term survival at lower than normal temperatures for a lowland population over a 19-day period (Gorman and Hillman 1977). The approximately 7**°** northward shift in latitude from Puerto Rico to Miami should result in a substantial change in the thermal climatic conditions experienced by lizards. Although differences in ambient temperature can be ameliorated by behavioral changes, body temperature in *A. cristatellus* is influenced by ambient temperature (Huey and Webster 1976; Hertz 1992). This suggests that a climatic shift should translate to body temperature differences between the native and non-native ranges, particularly in the winter when opportunities for thermoregulation are more limited. This study addresses two key questions: (1) how different are the thermal conditions in Miami compared to the tropical native range of *A. cristatellus* in Puerto Rico? and (2) does *A. cristatellus* show a phenotypic response to novel climatic conditions?

In this study, we used SDM and bioclimatic data to characterize the thermal niche shift of *A. cristatellus* from its native to its non-native range. We identified thermal variables that are outside the range of values experienced by native-range populations, indicating a niche shift. From these results, we generated hypotheses for adaptive phenotypic change for lower thermal tolerance, which we quantified by measuring the critical thermal minimum (CTMin) temperature. We predicted lower thermal tolerances and greater ability to acclimate to low temperature for *A. cristatellus* populations in Miami compared to those in Puerto Rico. We measured summer CTMin in field-caught *A. cristatellus* from Miami (the two introduced populations) and Puerto Rico (the two native-range source populations). For comparison to other anoles in Miami, we included the native species, *A. carolinensis,* and a long-term invader, *A. sagrei*. Using the same set of lizards, we conducted a low-temperature acclimation experiment to determine if adults showed short-term phenotypic plasticity in lower thermal tolerance. Finally, we measured winter CTMin in field-caught lizards from the same Miami populations to determine if field acclimatization was consistent with the results from the laboratory acclimation experiment.

## Materials and Methods

### Thermal niche modeling

To test for a thermal niche shift from the native to non-native range of *A. cristatellus*, we modeled habitat suitability in the native range of Puerto Rico and then projected this model to predict the species’ potential distribution in Florida using MaxEnt 3.3.3e (Phillips et al. 2006; Elith et al. 2010). We used georeferenced locality data from natural history museums obtained from HerpNet (accessed January 2011) and fieldwork conducted by the authors (native range *n* = 105 and non-native range *n* = 50). We evaluated 11 temperature-based variables (BIO 1–11) at 1-km^2^ spatial resolution from the WORLDCLIM 1.4 dataset (Hijmans et al. 2005) for inclusion in niche models. These bioclimatic data layers represent annual trends, seasonality, and extremes of temperature. Using data extracted at each locality, we generated a Pearson-product correlation matrix of these eleven temperature variables to identify and remove highly correlated variables (*r*≥ 0.85). This resulted in five remaining temperature variables: BIO 2, mean diurnal range (mean of monthly [maximum temperature – minimum temperature]); BIO 4, temperature seasonality (standard deviation × 100); BIO 5, maximum temperature of the warmest month; BIO 6, minimum temperature of the coldest month; and BIO 7, temperature annual range. We used the default modeling parameters for MaxEnt as suggested (Phillips et al. 2006). We mapped occurrence probabilities ranging from 0 to 1 for both Puerto Rico and Florida.

Potential difficulties modeling the distributions of range-shifting species are well known (see Elith et al. 2010); however, in this study our modeling objective is not to predict the non-native distribution per se, but rather to detect a shift in the thermal climate from the native to non-native range and, if such a shift exists, to quantify its direction and magnitude. In general, two outcomes are consistent with a climatic niche shift. First, extrapolation to novel climate space implicitly suggests a niche shift, although similarity between the new environments and those in the training sample must be evaluated (Elith et al. 2011). Second, a model that is trained in the native range and is transferable to the non-native range (i.e., within a similar range of climatic space), but results in zero or low occupancy probabilities, suggests a niche shift. In contrast, transferability and high occupancy probabilities in the non-native range indicate suitable climatic conditions similar to those in the native range, and therefore, lack of a niche shift. Portions of a species’ non-native distribution may fall into each of these categories.

We addressed the issue of transferability in two ways. First, we explored two options for delimiting the geographic extent from which background data (i.e., pseudoabsences) are drawn. MaxEnt modeling minimizes the relative entropy between the two probability densities estimated from the presence and background data (Phillips et al. 2006; Elith et al. 2011), and it is well established that the geographic extent from which background data are drawn affects occupancy predictions, model performance, and variable importance (e.g., VanDerWal et al. 2009; Anderson and Raza 2010). In general, as the study region increases in geographic extent, the model tends to overfit conditions near presence localities due to the increasing environmental differences between presence and background points, but also minimizes clamping (i.e., prediction in geographic areas with environmental values outside those used to train the model). In the first model, background data were drawn from the entire Caribbean basin, including the Greater and Lesser Antilles, northern South America, Central America, eastern Mexico, and Florida. We justify this choice because *Anolis* lizards exist throughout this area (Schwartz and Henderson 1991), showing the region has a suitable climate for anoles in general. In the second model, we restricted background data to the extent of presence locations on Puerto Rico, which includes the source populations for the two introductions to Miami (Kolbe et al. 2007). These two models span the range of appropriate backgrounds, allowing us to evaluate its effect on occurrence probabilities.

The second way we evaluated transferability was by using options in MaxEnt 3.3.3e that assess the extent of extra-polation using multivariate environmental similarity surfaces (MESS) and that identify the most dissimilar (MoD) variable in the projected space compared to the training range (Elith et al. 2010). We used MESS to measure similarity in the set of temperature variables used in the MaxEnt model between each 1-km^2^ cell in the non-native range of Florida and the distribution of values from the entire native range in Puerto Rico. Positive values indicate similarity in environmental space, whereas increasingly negative values show greater dissimilarity and, therefore, reduced transferability of the model. We interpret negative MESS values as evidence for a climatic niche shift between the ranges with increasingly negative values indicating shifts of greater magnitude. MoD identifies the variable furthest outside the range of training values from the native range in Puerto Rico for each 1-km^2^ cell in the non-native range of Florida. This “most dissimilar” variable is the climatic attribute that has shifted the most from the native to non-native range.

To complement the niche modeling analyses and compare the climate space occupied by native and non-native populations, we extracted data from presence points for the same five temperature variables (BIO 2, 4–7) used in the niche modeling. We tested for mean differences in the thermal climate space between the two ranges using multivariate analysis of variance (MANOVA) and analysis of variance (ANOVA) on individual variables. Variables were log transformed prior to analyses, which were all conducted in JMP 8 (JMP 1989–2009). These results were used both to describe the direction and magnitude of thermal niche change from the native to non-native range with reference to the importance of these variables in the thermal niche modeling and to make explicit predictions for divergence in thermal tolerance and thermal acclimation ability. We also compared the thermal climatic space occupied by *A. cristatellus* in the two non-native populations, South Miami (*n* = 37) and Key Biscayne (*n* = 9), and the two native-range source populations in the Agua Claras/Ceiba area (*n* = 6) and San Juan (*n* = 8).

### Population variation in critical thermal minimum

We compared six groups of adult male lizards to test for a difference in lower thermal tolerance among three *Anolis* species, including four populations of *A. cristatellus* (Table 1). We collected the two native *A. cristatellus* populations on April 8–9, 2010, *A. sagrei* on April 23–29, 2010, and *A. carolinensis* and the two introduced *A. cristatellus* populations on June 14–15, 2010. Introduced *A. sagrei* and native *A. cristatellus* collected in April 2010 were individually housed under shaded ambient weather conditions in South Miami until the start of the acclimation experiment, while *A. carolinensis* and introduced *A. cristatellus* were free living in the Miami area during this time. Thus, all lizards experienced similar weather conditions for approximately 8 weeks leading up to the experiment.

**Table 1:** Sites from which *Anolis* lizards were sampled for critical thermal minimum (CTMin) in Miami, FL and Puerto Rico. Native-range source populations are San Juan, PR for Key Biscayne, FL and Fajardo/Ceiba, PR for South Miami, FL

Population	Site	Range	Latitude (°N)	*N*
*cristatellus*	San Juan (SJ)	Native	18.4	20
*cristatellus*	Key Biscayne (KB)	Introduced	25.7	20
*cristatellus*	Fajardo/Ceiba (FC)	Native	18.3	14
*cristatellus*	South Miami (SM)	Introduced	25.7	20
*sagrei*	South Miami	Introduced	25.7	20
*carolinensis*	South Miami	Native	25.7	20

To assess lower thermal tolerance, we measured the critical thermal minimum (CTMin; Cowles and Bogert 1944; Spellerberg 1972). This widely used index of low-temperature tolerance in ectotherms is defined as the lower temperature at which an animal loses its ability to right itself. Our initial CTMin measurement for each lizard was taken on June 16, 2010. Starting from a body temperature (*T*_b_) of 22.1–27.2°C, we cooled lizards by placing them individually in small plastic containers inside an ice-filled cooler. We tested the righting response of each lizard after approximately 10 min by flipping it on its back and, if necessary, stimulating its venter with a small probe. *T*_b_ was taken at this time by inserting a thermocouple probe (30 gauge) approximately 5 mm into the cloaca. *T*_b_ was read on an Omega digital thermocouple thermometer (HH501DK, Type K). If the lizard did not right itself within 30 s, then the *T*_b_ was recorded as its CTMin. If the lizard did right itself, then it was further cooled and retested when showing signs of lethargy, approximately every 5–10 min. We also measured several covariates including body size (i.e., mass) and those relating to assay conditions (i.e., cooling rate [(starting *T*_b_– CTMin)/total time cooling], starting Tb, total time cooling, and time of day), which could potentially affect the measure of CTMin (Terblanche et al. 2007; Chown et al. 2009; Kolbe et al. 2010). Preliminary screening of these covariates revealed that only cooling rate significantly affected CTMin. We tested for a significant difference in CTMin among populations using analysis of covariance (ANCOVA) with cooling rate as a covariate, and used Tukey's honestly significant difference (HSD) post hoc test to determine which populations were significantly different. The interaction with the cooling rate covariate was nonsignificant and removed from the final model.

### Low-temperature acclimation experiment

To test for an effect of low-temperature acclimation on CTMin, lizards were housed under controlled conditions for 4 weeks. Ten lizards per population (except *A. cristatellus*– Fajardo/Ceiba; *n* = 7) were maintained on a natural light cycle, fed crickets 2–3 times per week, and misted twice daily. To simulate winter conditions, temperature was maintained at an average of 22.5°C (range = 21.8–23.6°C) over the 4-week acclimation period, which is within the normal range of temperatures for December–February in Miami (mean = 20.7°C, average high = 25.3°C, average low = 16.3°C). In contrast, over the same 4-week period in June–July 2010, ambient temperatures in Miami averaged 29.8°C (range = 22.8–35°C). We used repeated-measures ANCOVA with average cooling rate as a covariate to test for a between-subjects population effect (native *A. carolinensis*, introduced *A. sagrei*, and two introduced and two native *A. cristatellus* populations), and within-subjects effects of acclimation time (initial, 2 weeks, and 4 weeks) and the population-by-acclimation time interaction on CTMin. Interactions involving average cooling rate were nonsignificant and removed from the final model. We tested for a simple effect of acclimation time on CTMin for each population separately with paired *t*-tests comparing the initial and 4-week CTMin values. All populations experienced some mortality (not during the CTMin assays); thus, only those lizards remaining after 4 weeks were included in this analysis (mean = 8.2 lizards per population).

### Winter acclimatization of critical thermal minimum

An important assumption of our low-temperature acclimation experiment is that ambient winter temperatures in Miami elicit a similar acclimatization response in CTMin in free-living lizards; that is, the laboratory acclimation produces similar results to field acclimatization. To test this, we collected lizards in Miami on February 25–26, 2011, from the same localities as in the summer (*A. carolinensis* [*n* = 10], *A. sagrei* [*n* = 12], *A. cristatellus* [*n* = 12] from South Miami, and *A. cristatellus* [*n* = 10] from Key Biscayne). Winter temperatures in Miami for the 4 weeks prior to lizard collection averaged 22.2°C (range = 17.6–26.3°C), which is nearly identical to the mean temperature during the acclimation experiment. Lizards were held at ambient Miami conditions for several days, then 20.3–23.2°C on the day prior to measuring CTMin (March 2, 2011). We measured this winter CTMin using the same protocol as before, starting *T*_b_ ranged from 21.5°C to 24.8°C. To test for a seasonal acclimatization effect in CTMin for each species, we used a nested ANCOVA with population and season nested within population as fixed effects. These data included the winter CTMin values and previously collected summer CTMin values from June 16, 2010 (see previous section). Preliminary covariate screening revealed total cooling time was negatively related to CTMin. This covariate was included in the model, but interactions with the main factors were nonsignificant and removed from the final model. Tukey's HSD post hoc test was used to determine which populations differed significantly in CTMin between the summer and winter.

## Results

### Thermal niche modeling and thermal variable analyses

**Figure 2:**
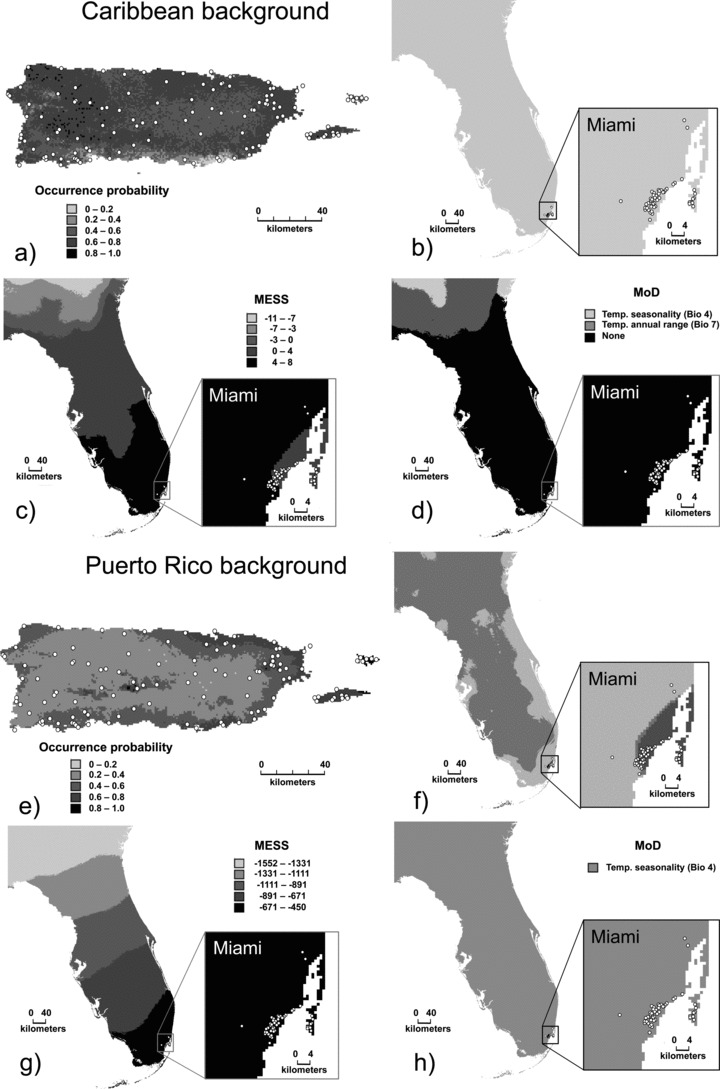
MaxEnt models of the potential distribution of *A. cristatellus* using two methods to define the study region. In method 1 (a–d), the entire Caribbean basin including the non-native range in Florida was used to train the model for native-range localities (a) and this model was projected to the climatic space in Florida (b). Occurrence probabilities from 0 to 1 are shown for both the native range of Puerto Rico and non-native range in Florida (a and b). We evaluated the extent of climatic extrapolation for the model trained on native-range localities and projected to the non-native range using multivariate environmental similarity surfaces (c), where increasingly negative values indicate more dissimilar climatic space. We also identified the most dissimilar climatic variable in the projected space compared to the training range (d). In method 2 (e–h), we limited the background for model training to the known localities on Puerto Rico (e) and then projected this model to the climatic space in Florida (f) with occurrence probabilities ranging from 0 to 1 (e and f). We again calculated the multivariate environmental similarity surfaces (g) and identified the most dissimilar climatic variable (h) for this model. White dots indicate locality points in the native and non-native ranges. Cell size equals 1 km^2^.

Thermal niche predictions using alternative backgrounds varied in model performance, importance of climatic variables, and degree of extrapolation, but both models predicted zero to moderate (i.e., ∼0.5) occurrence probabilities for the current non-native distribution of *A. cristatellus* in the Miami area (Fig. 2). The model using the Caribbean basin background had very good discrimination ability (AUC_training_ = 0.993, AUC_test_ = 0.991) and predicted occurrence probabilities of zero throughout Florida (Fig. 2b). Temperature seasonality (BIO 4; 65%) contributed most to the model, followed by maximum temperature in the warmest month (BIO 5; 13%), temperature annual range (BIO 7; 13%), and mean diurnal range (BIO 2; 9%), but minimum temperature of the coldest month (BIO 6) did not contribute to the model. Clamping was not observed, meaning thermal variable values in Florida are not outside the range of training values. Moreover, MESS and MoD indicated that no thermal variables in central or south Florida are outside the range of the training data (Fig. 2c and d). These results were consistent with expectations for models trained using backgrounds that draw from a broad climatic space (i.e., Caribbean basin) that includes values similar to where the model will be projected (i.e., Florida), and indicated the model is transferable (Elith et al. 2011).

In contrast, the model using only Puerto Rico for background points performed worse (AUC_training_ = 0.677, AUC_test_ = 0.669), but predicted moderate occurrence probabilities up to ∼0.5 in the Miami area (Fig. 2e–h). Temperature annual range (BIO 7; 43%) and minimum temperature of the coldest month (BIO 6; 36%) contributed most to the model, followed by maximum temperature in the warmest month (BIO 5; 10%), temperature seasonality (BIO 4; 8%), and mean diurnal range (BIO 2; 4%). Clamping was moderate for the Miami area, and negative MESS values in south Florida, which become increasingly so going northward in Florida, suggested dissimilar thermal values for non-native range points compared to the native range in Puerto Rico (Fig. 2g). In particular, temperature seasonality (BIO 4) values in Florida were the furthest outside the training range from Puerto Rico as identified by MoD (Fig. 2h), suggesting a substantial shift between ranges for this variable. Low to moderate occurrence probabilities for native-range models projected to Florida, and thermal variable dissimilarity and extrapolation for the model using the Puerto Rico background support a shift in the thermal niche of *A. cristatellus* during its introduction.

**Figure 3:**
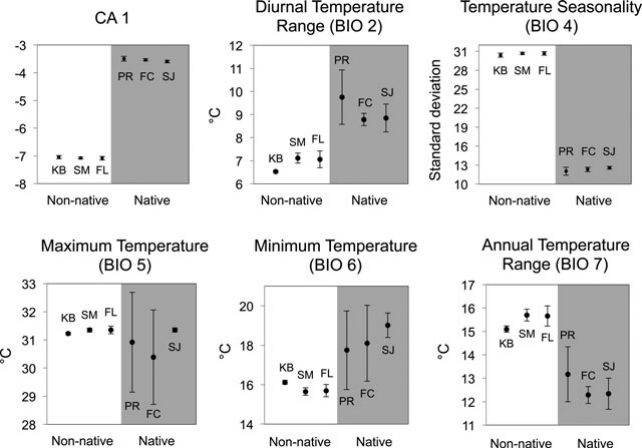
Differences in thermal variables used in the SDM between the non-native and native ranges of *A. cristatellus*. Mean (±1 SD) values are given for the two introduced populations, Key Biscayne (KB) and South Miami (SM), the non-native range in Florida (FL), the native range in Puerto Rico (PR), and the two native-range source populations, Fajardo/Ceiba (FC) and San Juan (SJ). The gray background indicates native range values. CA 1 is the first canonical axis from the MANOVA with all five thermal variables, including BIO 2, mean diurnal range (mean of monthly [maximum temperature - minimum temperature]); BIO 4, temperature seasonality (standard deviation); BIO 5, maximum temperature of the warmest month; BIO 6, minimum temperature of the coldest month; and BIO 7, temperature annual range.

Locality points from the non-native Miami and native Puerto Rican ranges of *A. cristatellus* differed significantly in the five temperature variables (Fig. 3; MANOVA: *F*_4,150_ = 4481.48, *P* < 0.0001). Follow-up ANOVAs showed highly significant differences between ranges in four thermal variables with non-native localities having a narrower mean diurnal range of temperature (BIO 2; *F*_1,153_ = 299.87, *P* < 0.0001, *R*^2^ = 0.66), greater temperature seasonality (BIO 4; *F*_1,153_ = 15560.69, *P* < 0.0001, *R*^2^ = 0.99), lower minimum temperature of the coldest month (BIO 6; *F*_1,153_ = 47.29, *P* < 0.0001, *R*^2^ = 0.24), and greater annual range of temperature (BIO 7; *F*_1,153_ = 179.08, *P* < 0.0001, *R*^2^ = 0.54), but no difference in maximum temperature of the warmest month (BIO 5; *F*_1,153_ = 3.41, *P* = 0.0667, *R*^2^ = 0.02). In particular, the large increase in temperature seasonality corresponded with the extrapolation detected for this variable in the thermal niche modeling (Fig. 2h). Despite the proximity of the two non-native populations in Miami (∼12 km), they differed significantly in thermal variables (MANOVA: *F*_4,41_ = 53.55, *P* < 0.0001) with the South Miami population showing a greater mean diurnal range of temperature (BIO 2; *F*_1,44_ = 67.99, *P* < 0.0001, *R*^2^ = 0.61), greater temperature seasonality (BIO 4; *F*_1,44_ = 10.68, *P* = 0.0021, *R*^2^ = 0.20), higher maximum temperature of the warmest month (BIO 5; *F*_1,44_ = 27.16, *P* < 0.0001, *R*^2^ = 0.38), lower minimum temperature of the coldest month (BIO 6; *F*_1,44_ = 47.51, *P* < 0.0001, *R*^2^ = 0.52), and greater annual range of temperature (BIO 7; *F*_1,153_ = 48.33, *P* < 0.0001, *R*^2^ = 0.52). These differences were small compared to those found between the native and non-native ranges; however, they consistently showed South Miami temperatures are more variable and lower than those of Key Biscayne. By contrast, the two native-range source areas in Puerto Rico did not differ significantly in the five thermal variables (MANOVA: *F*_4,9_ = 0.91, *P* = 0.4981; all univariate ANOVAs *P* > 0.10).

Thermal niche models and thermal variable analyses revealed how the thermal niche of *A. cristatellus* shifts from its native to non-native range, allowing us to make clear predictions for differences in thermal traits between native and non-native populations. First, the lower minimum temperature of the coldest month in Miami compared to Puerto Rico (Fig. 3) leads us to predict lower thermal tolerances for *A. cristatellus* in Miami than for those in Puerto Rico. A corollary to this prediction is that Florida's native species, *A. carolinensis*, should have the lowest thermal tolerance of the species studied, followed by the long-term invader *A. sagrei*, then the more recently introduced *A. cristatellus*, and finally the native-range *A. cristatellus*. Second, the higher temperature seasonality and annual temperature range in Miami compared to Puerto Rico (Figs. 1g, h and 2) predicts that Miami populations should be able to acclimate to lower temperatures. Temperate ectotherms show greater physiological acclimation abilities than tropical species (e.g., Feder 1982; Layne and Claussen 1982; Tsuji 1988). We predict that *A. carolinensis*, *A. sagrei*, and the two introduced populations of *A. cristatellus* will similarly acclimate to low temperatures by reducing their CTMin, but that the two native *A. cristatellus* populations will not respond to low-temperature acclimation.

### Population variation, low-temperature acclimation, and winter acclimatization in critical thermal minimum

**Figure 4:**
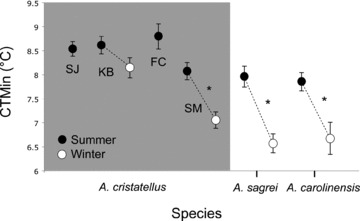
Mean critical thermal minimum (CTMin) temperatures for six *Anolis* lizard populations sampled from Miami, FL and Puerto Rico prior to the start of the low-temperature acclimation experiment. For *A. cristatellus* (gray background), the population pairs from San Juan (SJ)-Key Biscayne (KB) and Fajardo/Ceiba (FC)-South Miami (SM) correspond to the native source (Puerto Rico)-non-native recipient (Miami, FL) populations identified in Kolbe et al. (2007). Introduced *A. sagrei* and native *A. carolinensis* were sampled in Miami, FL. Dashed lines connect summer (black dots) and winter (white dots) measurements of CTMin for *A. cristatellus* (Key Biscayne, KB, and South Miami, SM), *A. sagrei*, and *A. carolinensis* to assess field acclimatization. An asterisk indicates a significantly lower CTMin value in winter based on Tukey's HSD post hoc test. Bars indicate ±1 SE.

After adjustment by covariate (cooling rate), field-caught *Anolis* lizards from populations in Miami and Puerto Rico differed significantly in their summer CTMin (Table 2a) with Tukey's HSD post hoc test showing only *A. carolinensis* has a lower CTMin compared to *A. sagrei* and the four *A. cristatellus* populations (Fig. 4). That *A. carolinensis* showed greater low-temperature tolerance than the other species is consistent with our prediction; however, the lack of difference among the other populations is unexpected.

**Table 2:** Results from ANCOVAs testing factors affecting critical thermal minimum (CTMin) among populations of *Anolis* lizards. Models are (a) ANCOVA with population (*A. carolinensis*, *A. sagrei*, and four populations of *A. cristatellus*) as a fixed effect and cooling rate as a covariate, (b) repeated-measures ANCOVA with the between subjects effect of population, within subject effect of acclimation time (initial, 2 weeks, and 4 weeks), and average cooling rate as a covariate, and (c) nested ANCOVA with season nested within population (summer and winter), population and total cooling time as a covariate

Factor	*F*	df	*P*
(a) Field-measured CTMin (*R^2^* = 0.281)			
Population	4.72	5,107	0.0006
Cooling rate	19.39	1,107	<0.0001
(b) Laboratory acclimation of CTMin			
Between subjects:			
Population	9.76	5,42	<0.0001
Average cooling rate	16.94	1,42	0.0002
Within subject:			
Acclimation time	6.27	2,41	0.0042
Population by acclimation time	3.29	10,82	0.0013
(c) Summer to winter field acclimatization of CTMin (*R*^2^ = 0.533)			
Season (population)	18.68	4,115	<0.0001
Population	8.22	3,115	<0.0001
Total cooling time	31.71	1,115	<0.0001

**Figure 5:**
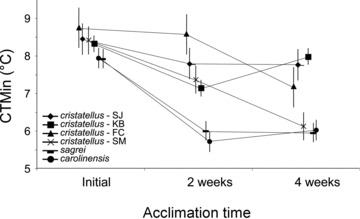
Mean critical thermal minimum (CTMin) temperatures at three acclimation times (initial, 2 weeks, and 4 weeks) for six *Anolis* lizard populations from the low-temperature acclimation experiment (mean temperature during acclimation = 22.5°C). *Anolis cristatellus* populations are abbreviated as follows: Key Biscayne (KB) and South Miami (SM) for the non-native range, and Fajardo/Ceiba (FC) and San Juan (SJ) for the native-range source populations. Lines are offset for clarity. Bars indicate ±1 SE.

In the laboratory acclimation experiment, repeated-measures ANCOVA revealed a significant difference in CTMin among populations, acclimation times, and their interaction while controlling for average cooling rate (Table 2b and Fig. 5). Starting from initially similar values (except for *A. carolinensis*), the 4-week acclimation period produced a significant decrease in CTMin of approximately 2°C for *A. carolinensis*, *A. sagrei*, and the South Miami population of *A. cristatellus* (Table 3 and Fig. 5). These results were consistent with predictions based on higher seasonality and annual range of temperatures in south Florida. By contrast, acclimation time was not a significant predictor for the other three *A. cristatellus* populations despite a nonsignificant trend of decreasing CTMin over the course of the experiment (Table 3). As predicted, the two native *A. cristatellus* populations did not show an ability to acclimate to low temperatures. Unexpectedly, the introduced population of *A. cristatellus* from Key Biscayne failed to acclimate to low temperature under the same experimental conditions.

**Table 3:** Results of paired *t*-tests between initial and 4-week CTMin values for the six populations of *Anolis* lizards. Data are limited to only those lizards that survived to the end of the low-temperature acclimation experiment (*n* = 49). The Bonferroni adjusted *P*-value was 0.0083 (Rice 1989). Significant *P*-values are bold

Population	Initial CTMin	4-week CTMin	*t*	df	*P*
	(mean ± SE)	(mean ± SE)			
*cristatellus*—SJ	8.4 ± 0.2	7.8 ± 0.4	−2.10	9	0.0646
*cristatellus*—KB	8.3 ± 0.2	8.0 ± 0.2	−0.80	6	0.4518
*cristatellus*—FC	8.7 ± 0.5	7.1 ± 0.5	−1.78	4	0.1491
*cristatellus*—SM	8.4 ± 0.2	6.1 ± 0.2	−7.17	7	**0.0002**
*sagrei*	7.9 ± 0.3	5.9 ± 0.3	−4.33	9	**0.0019**
*carolinensis*	7.9 ± 0.3	6.0 ± 0.3	−4.87	8	**0.0012**

SJ, San Juan; KB, Key Biscayne; FC, Fajardo/Ceiba; SM, South Miami.

When we compared summer and winter CTMin values, both population and season nested within population significantly affected CTMin in Miami *Anolis* lizards (Table 2c), after adjustment by covariate (total cooling time). Tukey's HSD post hoc test indicated *A. carolinensis*, *A. sagrei*, and the South Miami population of *A. cristatellus* decreased significantly in CTMin from summer to winter, but the Key Biscayne population of *A. cristatellus* did not (Fig. 4). This result was consistent with findings from the 4-week laboratory acclimation experiment (Fig. 5), albeit CTMin values after laboratory acclimation were slightly lower than winter CTMin values (Figs. 4 and 5). In summary, the Key Biscayne population did not acclimate in the laboratory experiment, nor did its winter CTMin value indicate seasonal acclimatization in the field, whereas the South Miami population of *A. cristatellus*, the long-term invader *A. sagrei*, and the native *A. carolinensis* all acclimated in the laboratory and reduced their CTMin seasonally.

## Discussion

Phenotypic change may facilitate the establishment and spread of invasive species, and could alter interspecific interactions, perhaps increasing negative impacts on native species (Strauss et al. 2006). Thus, an important aim of invasion biology is to identify the causes and consequences of phenotypic change during invasion (Sakai et al. 2001; Lee 2002; Sax et al. 2005). We detected a shift in the thermal climate for the lizard *A. cristatellus* during its introduction from Puerto Rico to Miami using SDM and thermal variable analysis (Fig. 3); consequently, we predicted that introduced populations would tolerate lower temperatures than native populations. However, only one of two introduced populations showed this predicted response, which was accomplished through the acquisition of low-temperature acclimation ability. Small climatic differences between the two introduced populations are consistent with this rapid acquisition of thermal plasticity, which may facilitate expansion of *A. cristatellus* outside of the Miami area into more thermally variable and colder regions.

### Evolution of low-temperature tolerance in the *A. cristatellus* invasion

Our SDM and analysis of bioclimatic variables agree that the thermal niche of *A. cristatellus* shifted during its introduction from Puerto Rico to Miami (Figs. 2 and 3), resulting in exposure to lower and more variable temperatures for lizards in Miami (Fig. 3). We interpret these changes as evidence for a shift in the realized thermal niche. Similar climatic niche shifts have been detected using correlative SDM in numerous studies, including other amphibian and reptile invaders in the Caribbean and southern United States (e.g., Greenhouse frog, *Eleutherodactylus planirostris* in Rödder and Lötters 2010; Brown anole, *A. sagrei* in Angetter et al. 2011). However, whether these shifts in the climatic conditions also lead to physiological changes in non-native populations (i.e., a shift in the fundamental niche) remains largely untested (but see Preisser et al. 2008) and should not be inferred from correlative models alone.

Within a relatively short period of time (∼35 years), *A. cristatellus* in South Miami acquired the ability to acclimate to low temperatures, similar to the native species, *A. carolinensis*, and the long-term invader, *A. sagrei* (Fig. 5). Our short-term acclimation experiment did not detect such plasticity in the native-range source populations in Puerto Rico (Fig. 5). This comparison provided the baseline for detecting the change in thermal plasticity, which would not be predicted for Miami populations based on data from the native-range sources (Fig. 5). We assume that the existing phenotypic variation in the source populations reflects that which was available at the time of introduction; that is, the distribution of genetic and phenotypic variation within the native-range sources has not changed substantially in the period between introduction in the mid-1970s and this study in 2010. In Puerto Rico, body temperatures of *A. cristatellus* vary seasonally and are influenced by ambient temperatures (Huey and Webster 1976; Hertz 1992). This suggests winter conditions in Miami should lead to lower body temperatures for lizards. If lower thermal tolerance is correlated with lower lethal temperature, as observed in other taxa (Hori and Kimura 1998; Das et al. 2004), and Miami populations of *A. cristatellus* are susceptible to low-temperature mortality as found in Puerto Rico (Gorman and Hillman 1977), then a basis for natural selection exists.

The increased thermal acclimation ability of the South Miami population may have been acquired by either developmental plasticity or adaptation of the acclimation response. Thermal environments experienced by embryos or hatchlings may affect the ability of adults to acclimate; for example, variation in egg incubation environments can affect behavior and phenotypes of hatchling lizards (Van Damme et al. 1992; Goodman and Walguarnery 2007; Goodman 2008; but see Warner et al. 2012). Although little is known about anole egg incubation environments in nature, it is an unlikely source of variation because climatic conditions in Miami and Puerto Rico broadly overlap during the reproductive season (Licht and Gorman 1970; Lee et al. 1989). Temperatures mostly diverge in winter when anoles are not reproductively active. In contrast, adaptive evolution is supported by the lower and more variable temperatures in Miami compared to Puerto Rico, and the divergence of the South Miami population from its native-range source population. However, more work, such as a laboratory common garden experiment, is needed to evaluate developmental plasticity and establish that differences in plasticity among populations are genetically based.

Although the South Miami population of *A. cristatellus* has clearly acquired plasticity for low-temperature tolerance during invasion, at least three nonmutually exclusive mechanisms could explain the difference between the two introduced populations in Miami. We have not evaluated the role of genetic drift, but we made explicit predictions of phenotypic change based on our modeling and thermal variable analyses, and these predictions are supported for thermal tolerances of the South Miami population. Therefore, we consider drift alone to be an unlikely mechanism underlying the shift in phenotypic plasticity observed in the South Miami population.

First, the South Miami population may have had more time to adapt to its new thermal conditions. This is unlikely as the two introduced populations were initially detected at similar times in the mid-1970s.

Second, the South Miami population could have greater additive genetic variance for low-temperature acclimation, which would lead to a greater phenotypic response to similar selective pressures. Molecular genetic variation within introduced populations is strongly influenced by the size of the propagule introduced and the number of introduction events, including those from genetically distinct sources (Dlugosch and Parker 2008). These factors also likely influence the amount of genetic variation underlying quantitative traits (Bacigalupe 2008; Lee and Gelembiuk 2008), such as plasticity in thermal tolerances. For *A. cristatellus* in Miami, each population is derived from a single source; however, there is evidence that the South Miami propagule was larger. Kolbe et al. (2007) detected four unique haplotypes in the South Miami population (out of 14 individuals sampled), whereas no haplotypic variation existed in the Key Biscayne population (out of nine individuals sampled). Haplotype diversity was similar in the two source populations, with all 11 individuals sampled in the San Juan area having unique haplotypes and 13 of 15 individuals having unique haplotypes in the Agua Claras/Ceiba area. In the absence of mutation, this evidence suggests that at least four females were introduced to South Miami and one female to Key Biscayne. Thus, a larger propagule in South Miami could have more additive genetic variance for low-temperature tolerance and thus have shown a greater response to selection (Lee 2002; Bacigalupe 2008). Future studies with more variable biparentally inherited markers (such as microsatellites) will allow for more accurate estimates of propagule sizes, but the hypothesis of different amounts of genetic variation cannot be rejected.

Lastly, the thermal environments between South Miami and Key Biscayne could differ. While this initially seemed implausible given the proximity of the two populations (∼12 km apart), our analysis of the same thermal variables used in the SDM reveal significant climatic differences between the two sites, such that the South Miami population experiences lower minimum and more variable temperatures (Fig. 3), which is consistent with the acquisition of low-temperature acclimation ability. Both localities are a mix of residential, commercial, and parklands with no obvious differences beyond Key Biscayne being an island. Thermal differences in the environment may be accentuated or dampened depending on individual behavior of lizards within each population, and it is difficult to predict lizard body temperatures for each location. Furthermore, the thermal differences between the two introduced populations are only a small percentage of the differences between Puerto Rico and Miami. Nonetheless, the thermal differences observed between populations in this study suggests further study of genetic and environmental factors may be warranted for understanding the acquisition of thermal plasticity in the *A. cristatellus* introduction.

### Phenotypic plasticity and invasion success

Phenotypic plasticity is often put forth as a trait that would facilitate invasion of novel environments, including exotic species invasions (Sakai et al. 2001; Lee 2002; Whitney and Gabler 2008). However, we rarely have the opportunity to test whether plasticity is needed for invasion success (Richards et al. 2006). A test of this proposition would require populations or species that vary in their capacity for a plastic response. Introduced populations of *A. cristatellus* show variation in their thermal acclimation ability, but both populations have persisted in Miami for similar time periods. The Key Biscayne population of *A. cristatellus* illustrates that low-temperature acclimation is not required for the establishment or persistence of this species in some parts of Miami, at least over the past 35 years. Similarly, low-temperature acclimation ability may not have been required for the establishment of the South Miami population, given the lack of plasticity in its native-range source population. However, its persistence may have been facilitated by the acquisition of thermal plasticity. We predict that this population will be able to spread farther north into colder and more variable thermal environments than the Key Biscayne population, although other factors may limit its spread in particular areas. The Key Biscayne population is also on an offshore island, which restricts dispersal and thus confounds any comparison of extent of geographic expansion.

### Species distribution modeling and thermal climate shifts in invasions

Some have suggested that the lack of climate match between a species’ native and introduced ranges is a good indication that a species is unlikely to become invasive (see Mandle et al. 2010 for discussion and references), but this argument can only be supported if using methods that accurately predict occurrence in the non-native range. Here, models trained in the native range and projected to the non-native range perform poorly in this regard; they predict zero to moderate occurrence probabilities in Florida despite the presence of *A. cristatellus* in Miami since the mid-1970s (Fig. 2). These results suggest caution should be used when drawing conclusions about the ability of an invasive species to become established or spread in the non-native range from SDM alone. If accurate occurrence probabilities in the non-native range are the primary goal of a study (Whitney and Gabler 2008), then methods that account for the fundamental niche and phenotypic divergence in non-native populations are needed.

The low occurrence probabilities from SDM for *A. cristatellus* in Miami may be due to several factors. First, models may better reflect the realized thermal niche of *A. cristatellus* in Puerto Rico (in part due to dispersal limitation) rather than its fundamental niche (Jackson and Overpeck 2000). That is, native-range *A. cristatellus* may have the capacity to tolerate lower temperatures than they experience in Puerto Rico, but only in cases such as introductions do they actually experience these conditions. If no change in thermal tolerance is observed for non-native populations, then our interpretation is that the population is accessing a portion of its fundamental niche not available in its native range. This appears to be the case for the Key Biscayne population (Fig. 4), which can tolerate the lower and more variable temperatures in Miami compared to Puerto Rico, but its thermal tolerance has not changed in order to accomplish this. Second, phenotypic change due to adaptation and/or plasticity in non-native populations may extend thermal tolerances beyond that of their native-range source population. This is supported by the acquisition of lower thermal tolerance acclimation in the South Miami population, which extends the fundamental niche beyond that of *A. cristatellus* in its native range.

## Conclusions

We integrated modeling, empirical, and experimental approaches to understand how thermal tolerances respond to changing climatic conditions during the *A. cristatellus* introduction. By comparing introduced populations to their native sources, we revealed rapid phenotypic change in thermal tolerances due to the acquisition of plasticity (Fig. 5), which was consistent between laboratory acclimation and field acclimatization (Fig. 4). We detected distinct trajectories for phenotypic change in independently introduced populations despite their proximity in Miami. Further study is needed to clarify the cause of this differential response; however, this result cautions against treating populations across the non-native range of an invader as homogeneous. Instead, it emphasizes that environmental, genetic, and phenotypic variation exists among non-native populations (e.g., Kolbe et al. 2007; Keller et al. 2009), which may influence evolutionary dynamics and have important consequences for establishment, spread, and impact on native species.
